# Longitudinal multiple case study on effectiveness of network-based dementia care towards more integration, quality of care, and collaboration in primary care

**DOI:** 10.1371/journal.pone.0198811

**Published:** 2018-06-27

**Authors:** Anke Richters, Minke S. Nieuwboer, Marcel G. M. Olde Rikkert, Rene J. F. Melis, Marieke Perry, Marjolein A. van der Marck

**Affiliations:** 1 Radboud university medical center, Donders Institute for Brain Cognition and Behaviour, Department of Geriatric Medicine, Nijmegen, The Netherlands; 2 Radboud university medical center, Radboudumc Alzheimer Centre, Nijmegen, The Netherlands; 3 Radboud university medical center, Department of Geriatric Medicine, Nijmegen, The Netherlands; 4 Radboud university medical center, Radboud Institute for Health Sciences, Department of Geriatric Medicine, Nijmegen, The Netherlands; University of Haifa, ISRAEL

## Abstract

**Introduction:**

This study aimed to provide insight into the merits of DementiaNet, a network-based primary care innovation for community-dwelling dementia patients.

**Methods:**

Longitudinal mixed methods multiple case study including 13 networks of primary care professionals as cases. Data collection comprised continuously-kept logs; yearly network maturity score (range 0–24), yearly quality of care assessment (quality indicators, 0–100), and in-depth interviews.

**Results:**

Networks consisted of median nine professionals (range 5–22) covering medical, care and welfare disciplines. Their follow-up was 1–2 years. Average yearly increase was 2.03 (95%-CI:1.20–2.96) on network maturity and 8.45 (95%-CI:2.80–14.69) on quality indicator score. High primary care practice involvement and strong leadership proved essential in the transition towards more mature networks with better quality of care.

**Discussion:**

Progress towards more mature networks favored quality of care improvements. DementiaNet appeared to be effective to realize transition towards network-based care, enhance multidisciplinary collaboration, and improve quality of dementia care.

## Introduction

Chronic conditions like dementia pose a great challenge to health care systems.[1] Primary care for community-dwelling dementia patients is multifaceted, especially in later stages in which the disease affects many aspects of the lives of patients and their informal caregivers. Medical issues fall under responsibility of the primary care physician (PP), but many patients also require other forms of care and support such as home care, nursing care, and temporary involvement of allied health professionals. Subsequently, patients often require case management to ensure continuity and availability of services, and primary care professionals are increasingly urged to work in a multidisciplinary manner.

In Dutch primary care system (Box 1), various care professionals are involved in care for community-dwelling people with dementia including medical disciplines (primary care physician, elderly care physician), care disciplines (community nurse, case managers), and social disciplines (social workers, caregiver supporters), who often work at different organizations. All Dutch inhabitants are registered at a primary care practice (PCP). Patients often have the same PP over many years resulting in long, trusting relationships. The PP has a gatekeeper function, has a generic perspective, and continues to be a key professional in the care for people with dementia. The Dutch care system is adapting to major policy changes, including widespread closing of elderly homes, a move towards a participatory society with incentives to stay at home longer, and stimulation of market mechanisms in health care. These trends have resulted in fragmentation, lack of expertise on dementia and multi-morbidity among primary care professionals, and unintended regional variation in care–characteristics that also exist in many other high-income countries.

**Box 1. Primary care in the Netherlands**

**Primary care for community-dwelling dementia patients in the Netherlands**
- Community-dwelling dementia patients receive care from multiple care professionals, including medical disciplines (primary care physician, elderly care physician), care disciplines (community nurse, case managers), and social disciplines (social workers, respite care workers).
- All Dutch inhabitants are registered at a primary care practice in close vicinity to where they live. Primary care physician referral is needed for specialist care. Indications to obtain home care are provided by municipalities or district nursing organizations.
- All Dutch inhabitants are obliged to have health care insurance and are free to choose between various private health care insurance companies. There is fragmentation in finances of services: Primary care, home care and nursing care are part of insurance and are paid for directly by private health care insurance companies; the organization and financing of social care is the responsibility of municipalities; case management is paid for by insurance companies, and exists in multiple formats and may be independent or part of home care organizations.
- Several national guidelines and documents are available on primary dementia care arrangements in the Netherlands, including guidelines for the primary care practice, a national standard for multidisciplinary dementia care, and agreements describing collaboration between the primary care practice and home care and elderly care physicians. Despite availability, uptake of and compliance with these documents in practice is low.
- Dementia care on a local level is determined by national, regional and local policies as well as existing facilities and by individual initiatives undertaken by the healthcare professionals. As a result, services and quality of local care are highly variable throughout the Netherlands.

National and international efforts to improve dementia care have shown some promising results[2, 3], but room for improvement remains. Interventions targeting specific suboptimal aspects of the care system so far lacked effectiveness because they did not comprehensively improve the integration and continuity of dementia care.[4, 5] For instance, interventions targeting care coordination, early diagnosis or educational interventions aimed at expertise of primary care physicians did not lead to desired outcomes.[4, 6] It is likely that the health care system itself requires adaptation as well, since improvement of individual components is not effective in improving dementia care.

This insight led to the development of DementiaNet. DementiaNet is an innovative primary dementia care approach that targets the transition towards network-based primary care by forming networks of professionals, who are supported in their change efforts.[7] It embodies a complex health care innovation, given the multiple interacting components of the program, the required behavioral changes of professionals, and the high degree of flexibility required to adapt to different local circumstances.[8] The implementation context is also highly complex, as it involves many different stakeholders (e.g. care professionals, municipalities, insurers, government). This high degree of complexity results in unpredictability of the expected changes and warrants an appropriate perspective on its evaluation. Hence, instead of asking whether an intervention works, evaluation should be aimed to identify if and how it contributes to reshaping a system in favorable ways.[9] In order to gain insight into such effects and to facilitate evidence-based primary care, an evaluation study was performed. This study aimed to answer the following questions: what are the merits and drawbacks of the DementiaNet approach; how are these achieved; and which factors influence these processes?

## Methods

### DementiaNet networks

DementiaNet encompasses a transition towards high quality, network-based care organized at a local level. The program’s key strategy is practice facilitation, a promising approach to supporting care redesign in which trained facilitators support primary care professionals.[10, 11] Multidisciplinary networks of primary care professionals are formed, which jointly provide care to a number of dementia patients. Desirably, networks include at least one professional of the medical (e.g. PPs), care (e.g. community nurses) and welfare (i.e. social workers, case managers) discipline. Inclusion of professionals is defined by the networks themselves and tailored to local sources and needs. As a consequence, each network is different in terms of size, represented disciplines, starting level of collaboration and quality of care.

The DementiaNet program consists of fixed and tailored elements.[7] The following four key components were applied in each network. Firstly, a transition towards network-based care was initiated, aimed at structural instead of ad hoc collaboration. According to a contingency approach on interprofessional practice typology, network-based care is a type of structured collaboration in which coordination is the most essential component. This is most suited for the primary care setting, where clinical work is more predictable and less urgent than in a hospital setting.[12] Secondly, one or two professionals in each network took on the role of network leader and were supported in this leadership role via individual and group coaching. This coaching enabled them to stimulate and facilitate multidisciplinary collaboration and improvement actions. Coaching was performed by two experienced trainers with backgrounds in primary care, who were trained on the job by experts on interprofessional learning. Thirdly, networks followed the Plan-Do-Check-Act method for quality improvement based on jointly identified improvement goals. Fourth, interprofessional training and practice-based learning were used to increase knowledge and competencies on dementia care and multidisciplinary collaboration. Since each network and their context varied, various aspects of DementiaNet were tailored to the local setting and needs, including actual improvement goals and plans, content of the interprofessional training and extent of leadership coaching. The DementiaNet networks provided care for the all community-dwelling dementia patients registered at the PCPs in the networks. Care tasks start when first signals of cognitive decline are present and professionals are contacted, throughout diagnostic process, and continues until a patient is in the later stages up until institutionalization in a care facility or death.

The enrollment of networks in the DementiaNet program was aimed at early adopters. Through various media, the start of the program was announced and motivated primary care professionals were invited and supported to initiate the formation of a local network.

### Study design

To fit the complexity of DementiaNet as a multi-faceted innovation embedded in a complex health care setting, a longitudinal mixed methods multiple case study was chosen (further elaborated in study protocol[13]). Each DementiaNet network served as a case with follow-up up to 24 months. This design fits the description of comparative case studies, in which qualitative and quantitative data are collected from multiple sources and integration of both types of data is performed to leverage the strengths of both.[14] Joint interpretation allows new insights to arise, beyond the information gained from separate sources.[15, 16] The study protocol was submitted for review to the medical ethics committee region Arnhem-Nijmegen, and they declared that formal judgment was not required (protocol number: 2015–2053). No informed consent was required.

### Data collection

Multiple sources of data were collected during the study period study period (January 2015–July 2017) for all networks: logs were kept continuously for network narratives, quantitative data was collected at baseline and yearly, and qualitative data after 12 months.

#### Network narratives

A log on each network was kept with characteristics of the network and members, including the number of professionals and disciplines involved. Also, data was collected on the formation of the network and change efforts, with information on process and actions undertaken before enrolment; on collaboration at baseline and changes over time; on improvement goals, actions and achievements as part of quality improvement; and any specifics that may influence development over time.

#### Network maturity

As part of yearly assessments, structured interviews were held with network leaders with topics based on the Rainbow Model of Integrated Care to assess network maturity.[17] The global network maturity score (range 0–24) was derived by rating 8 items (population-based care, person-focused care, clinical integration, professional integration, organizational integration, system integration, functional integration, normative integration) on a scale with predefined levels (score 0–3). Rating was performed independently by two researchers (AR,TK), after which consensus was reached on each item. Higher scores indicate higher maturity. No psychometric properties of this score are reported in literature yet.

#### Quality of care

Prior to the study, an expert panel developed a set of 13 quality indicators (QIs) based on available dementia care guidelines and agreements, which was pilot-tested by primary care professionals for feasibility, relevance, and comprehensiveness.[13] Prior to analysis, baseline scores on the initial set of QIs were reviewed for appropriateness, taking into account feedback from the networks, missingness, floor and ceiling effects, and coherence with definitions. This led to a final, more concise set of six QIs: proportion of patients with (1) involvement of case management; (2) dementia diagnosis in primary care setting; (3) recent geriatric assessment; (4) recent consideration during a multidisciplinary meeting; (5) recent polypharmacy check; and (6) average number of emergency consultations per year.

Data on these QIs were reported for shared dementia patients of each network and collected yearly via a registration document, which was completed by a network member based on information as registered in electronic patient files. Sum score were constructed by averaging scores on each indicator, yielding a total score between 0 and 100 (higher scores indicating better quality).

#### Experiences and perspectives

Semi-structured interviews were held with professionals to obtain insight into experiences with and perspectives on DementiaNet, until data saturation was achieved. A purposive sample of professionals (n = 9) from networks that had been participating for at least one year were invited for interviews, securing input from multiple networks and different disciplines. A trained qualitative researcher (IM) performed the interviews using a topic list and performed a member check after the interviews.

### Analyses

Quantitative analyses were performed in R (package lme4). Interview transcript analysis was performed in Atlas.ti.

#### Quantitative analysis

For all 13 networks, the global network maturity score and quality of care sum score were calculated at start and 12 months and for 6 networks also at 24 months. These scores were used for quantitative analysis to assess overall changes in network maturity and QI scores over time by means of mixed regression models to account for repeated measures (random intercepts per network, fixed effect for time). Association of network maturity with QI scores was assessed.

#### Qualitative analysis

Logs were processed into narratives of each network by three researchers (AR, MN, MP). Transcripts from the semi-structured interviews were independently coded by two trained researchers (AR, IM), after which both coding schemes were jointly reviewed to reach consensus. Subsequently, codes were categorized to identify major themes. Quotes belonging to each major theme were independently reviewed to draw overall findings per theme, after which a consensus round yielded the overall findings.

#### Integration

Trends on network maturity and QI scores of each individual network were jointly considered with narratives of each network, in order to identify possible explanations for (lack of) change. Both qualitative and quantitative data were presented in a joint display (table) that simultaneously arrays the quantitative and quantitative results, in order to integrate the data by bringing the data together through a visual means to draw out new insights beyond the information gained from the separate quantitative and qualitative results.[14] Networks with similarity in specific aspects of quantitative data were identified and compared based on the narratives to explain patterns, i.e. comparisons prompted by quantitative patterns. Such comparisons included: networks with comparable but low starting levels of network maturity, with some showing enormous increase and others only minor increase, networks with improvement in network maturity, with some showing accompanied improvement in quality of care and others not, and networks with declining levels of quality of care. Networks were grouped based on common characteristics to explore the influence on trends in quantitative measures, i.e. comparisons prompted by characteristics and narrative patterns. Characteristics that were considered included: whether it concerned a new or existing collaboration, the level of collaboration upon start in the study, the type of improvement goals, strength of leadership, the catchment area size of the networks, and size of the network. Also, networks with highly promising experiences (best practices) and networks that have failed were reviewed more in-depth. Additionally, findings of quantitative analysis in combination with narratives were compared to the findings from semi-structured interviews to identify convergence or divergence among topics and to identify how these complemented each other. Integration was carried out based on consensus (AR, MN, MP, MvdM) and verified by the other authors.

## Results

### DementiaNet networks

Seventeen networks started between January 2015 and June 2016. Four of them ceased active participation within the first year. Reasons were either related to lack of intrinsic motivation (e.g. participation was initiated by local government) or lack of time, resulting in insufficient momentum for a transition process. Hence, results refer to 13 networks.

The median number of professionals in the networks was 9 (range 5–22). The composition regarding disciplines varied, with PPs, practice nurses, case managers, and community nurses being most represented. Eleven networks included professionals from medical, care, and welfare disciplines. All networks were followed for at least one year, and six for two years, resulting in total in 19 yearly evaluations. A detailed description of each network’s characteristics and proceedings is described in Table 1.

**Table 1:** Characteristics and narrative summaries of the primary care networks in the DementiaNet program.

Network	Compo-sition at start	Number of network members (number of disciplines)	Collaboration	Network leaders	Catch-ment area	Network maturity score (start; year 1; year 2)	Quality of care score (start; year 1; year 2)	Caseload of patients (start; year 1; year 2)	Improvement goals (year)	Narrative summary
A	1 CM; 2 CN;1 GS; 1 PP; 1 PN.	6 (5)	Existing collaboration	CM, PP	Small	23.0; 25.5; 24.0	89.7; 94.8; 94.7	13; 17; 22	Working agreements on detection of cognitive decline (year 1) Increasing efficiency of care (year 2)	Started as a compact network where a lot of structures were already in place. It could be considered a best practice network from the start. They already participated in an elderly program and had a very well-functioning multidisciplinary meeting and high levels of collaboration at start. With highly dedicated network leaders and all the relevant professionals present in the network, improvement plans were carried out well. A care map was constructed defining everyone’s role in dealing with signals of cognitive decline. In the second year, more insight was obtained in everyone’s care tasks to improve efficiency by removing doubly executed tasks and identifying gaps. Even though collaboration was already high and structured at start, bonds between the professionals were strengthened over the two years of participation by yearly evaluation of their collaboration.
B	2 CM; 3 CN; 1 GS; 1 OT; 3 PP; 1 PT; 2 WF; 1 other.	13 (8)	Existing collaboration	CM, CN	Large	12.0; 14.0; 14.5	45.6; 50.6; unknown	19; 16; unknown	Obtaining overview on “who does what” (year 1) dementia-friendly society (year 2)	In this neighborhood, a collaboration of several professionals was already in place. They decided to enroll in the DementiaNet program to get support in improving integrated care. They were mainly interested in improvements on a neighborhood-level instead of improving care for individual patients. A social care map was introduced. With lack of a strong position of the network leader, improvements were less prominent than potentially could have been. Also, considerable changes to the network took place, with two out of three PPs leaving and the primary network leader changing jobs.
C	1 CM; 9 CN; 2 GS; 3 MM; 1 PP; 1 PN; 3 WF; 1 other.	22 (8)	Existing collaboration	PP, PN	Large	14.0; 16.0; 15.0	71.4; 71.8; 76.9	35; 25; 30	Improvement of detection cognitive decline (year 1) geriatric assessments (year 1) introducing multidisciplinary meetings (year 2) multidisciplinary care plans (year 2)	A rather large network of care professionals for dementia was already established for several years, after the municipality and several care professionals actively recruited care professionals to work together. After several years, the municipality could not provide support anymore. Therefore, the network enrolled in the DementiaNet program to get support and guidance in improving care and to get training and education. The first year went well, with sufficient meetings and training, resulting in concrete agreements on care on cognitive decline detection. During the second year, some personal struggles caused interprofessional frictions among network members, with not everyone getting along and feeling included, but this was resolved. A multidisciplinary meeting was introduced and agreements were made. Additional training on geriatric assessment topic took place.
D	1 CM; 4 CN; 1 MM;2 PP; 2 WF.	10 (5)	New collaboration with unacquainted members	2 CNs	Large	8.5; 9.0; 11.5	41.1; 47.2; 40.0	15; 12; 9	Improving communication among professionals (year 1) uptake of digital communication tool (year 2) improvement of dementia expertise (year 2) getting welfare involved (year 2)	The initiative to participate came from a home care organization. The DementiaNet team helped to get the group of PPs on board; several other disciplines joined. During the first year, the network focused on implementation of a scan of the informal care network of each patient; however, most of the actions to be undertaken were dependent on the network leaders, as other network members took a passive role in the process, resulting in suboptimal improvement actions. Educational sessions on dementia content were also held. A start was made with ICT communication tools. The main effort of the second year was to move forward with the uptake of the ICT tool, which eventually proceeded steadily after some technical difficulties. Training sessions were evaluated positively and many involved professionals participated. However, enthusiasm of the network leaders who were the driving force decreased, due to lack of activity from other members in the first year. During the second year, the PP was succeeded by a new one, taking some time in getting all care processes on track. Also, both network leaders were absent for some time due to personal reasons. While some other parties remained in the network, they were represented by new members.
E	1 CM; 2 CN; 2 GS; 2 PP; 1 PN.	8 (5)	Relatively new collaboration	CM, PN	Small	10.0; 12.0; 14.0	42.9; 77.8; 68.5	7; 9; 13	Improvement of multidisciplinary meeting (year 1) improvement of signaling cognitive decline (year 1) increase expertise for dealing with problematic behavior (year 2)	The network formation was initiated by the network leaders who were aware of the fact that two of the PPs they worked with had difficulties in caring for dementia patients, which they experienced as well. They worked together on a patient-basis before without being a formal network. They started of small, but with the most important players involved. During the first year, their efforts in the context of the program have led to slowly but steadily accomplishing the first improvement goals, but mostly resulted in being more acquainted with each other and more and better communication between them, specifically between the case manager and PPs. The actions have also lead to a better overview of the population. During the second year, the actions aiming to improve their new goals revealed many differences in vision on better care. This resulted that most actions had to first be aimed at solving those discrepancies and less on improving actual care.
F	1 CM; 1 CN; 2 PP; 2 PN; 1 other.	7 (5)	Relatively new collaboration	CM, PN	Large	9.0; 13.5; 16.5	48.2; 59.2; 79.7	12; 21; 31	Improvement of multidisciplinary meeting (year 1) increasing expertise in diagnostics (year 1) increasing expertise on dementia (year 2) improving collaboration (year 2)	Before start, the members of this compact network already shared patients but no formal collaboration existed beyond ad hoc interactions. However, they felt there was a lot of room for improvement, as they did not feel fully competent on all aspects. Also, a new PP had just taken over the involved practice, covering many elderly patients. Support in tackling some issues was wanted. Over the first year, several trainings were held and improvement goals were considered to be achieved. Moreover, the network leaders were much more confident in their role, as viewed by themselves and others. During the second year, the PPs were better equipped and confident in diagnostics, and numbers of new diagnoses further increased. Next to training on the new topics of the improvement goals, they also initiated meetings for other disciplines (e.g. occupational therapists) to explain what they can offer in the care path of dementia patients, to become more acquainted with all involved professionals and thus enhance collaboration on a patient-level. The network composition was stable and the network showed to be capable of fairly independent improvement initiatives.
G	7 CN; 1 OT; 1 PT.	9 (3)	New collaboration with unacquainted members	2 CNs	Large	10.5; 9.0	70.8; 60.8	4; 5	Dealing with early signals of cognitive decline (year 1) introduction of multidisciplinary care plans (year 1)	This network was initiated by the local team of community nurses. They shared patients with a number of PPs but could not get them on board of the network prior to participation in the program. An occupational therapist and physiotherapist were interested in joining the network. The plan was to improve collaboration with PPs and case managers first, and have them join the network later. The community nurses often pick up signals of cognitive decline and suspect dementia, but there are no agreements on how to communicate these signals with the PP and how to make sure the patient is evaluated. Training was given to the network, which managed to get case managers involved. It improved the collaboration between community nurses and case managers, but the network was unable to get PPs involved. Training on multidisciplinary care plans did not take place because the network could not arrange a time for it.
H	1 CM; 2 CN; 2 PP; 1 WF.	6 (4)	Existing collaboration	CN, PP	Small	19.0; 22.5	40.7; 75.5	28; 28	Inclusion of welfare disciplines (year 1) improvement of dealing with complex care situations (year 1)	Right after initial enrollment in the program, the network leader became absent due to personal reasons and could not return. Therefore, actual start took place a year later, even though it remained unclear who formally took over the roll as network leader. It is a very concise network of people who had already worked together for many years but only included the core disciplines (PP, CN, and CM). In the year between the first attempt at enrollment and actual enrollment, they successfully included welfare workers in their multidisciplinary meetings. The year in the program started slow but eventually a lot of content training on complex care situations took place and agreements were made. Note: only data from the actual year in the program are used in the quantitative analysis.
I	1 CM; 6 CN; 1 OT; 2 PP; 4 WF; 2 other.	16 (6)	New collaboration	CN	Large	9.5; 10.0	53.6; 54.4	28; 25	Obtaining overview on “who does what” regarding dementia care to identify doubling and gaps (year 1)	For this network, the DementiaNet practice facilitators were contacted by a third (national) party with the intention to make this neighborhood ‘dementia-friendly’. The program team contacted multiple care professionals in this area with a shared patient caseload and eventually a network was formed. The majority of involved professionals were not really acquainted with each other at this point; hence this formed the improvement goal. This was successfully carried out, which led to more insight in the network for involved professionals and better information provision to patients and informal caregivers. The process has led to more connection among professionals.
J	1 CM; 1 CN; 1 MM;1 PP; 1 PN.	5 (5)	New collaboration with unacquainted members	CN	Small	10.5; 16.5	52.8; 56.3	18; 16	Obtaining overview on “who does what” regarding dementia care (year 1) improvement of diagnostic process (year 1)	A community nurse undertook the initiative to set up a local network, which was quickly formed. This was a compact network with only key players in dementia care, yet they were unacquainted with each other at enrollment in the program. Educational sessions were followed and these did not only increase expertise on the topic, but also greatly enhanced the connection between different professionals because they got to know each other much better. This also resulted in a better overview on each other’s tasks and skills and a social care map was constructed successfully. This network started at the very beginning by getting to know each other, but towards the end got around to working on actual care processes, which will be the main focus after the first year. The network is enthusiastic and stable with an active leader.
K	2 CM;2 CN;1 MM;1 OT;1 PT;2 WF;3 other.	12 (7)	New collaboration with unacquainted members	OT, WF	Small	10.5; 16.5	45.1; 50.0	8; 11	Social care map (year 1) increasing dementia expertise (year 1)	Initiative to participate came from a manager of a home care organization. A meeting was set up with the care professionals and after they expressed interest, other interested professionals were found from the primary care practice, day care and welfare. At enrollment, they were mostly unacquainted with each other. Their focus was to get to know each other and to get more insight into each other’s roles and tasks. This was highly stimulated by the network leader who showed to be skilled in connecting people. Interprofessional training further stimulated this and simultaneously increased expertise on complex care situations. Agreements were set out for patient care between involved professionals. It is a stable and compact network and attracted other interested professionals during the year.
L	1 CM; 8 CN;2 PP.	11 (3)	New collaboration	CM, CN	Small	12.0; 16.5	39.9; 87.7	22; 30	Introduction of multidisciplinary meetings (year 1) geriatric assessment (year 1)	Case manager initiated the formation of a network, by talking to several professionals in the area; primary care physician and home care joined. This network operates in a small village with only one home care organization, resulting in much overview. All key players are present in the network and highly involved; network leaders are enthusiastic and capable of undertaking action. They have implemented several initiatives on their own to move forward with the network, such as a comprehensive approach to formulating a vision and tackling possible discrepancies among network members. Improvement of multidisciplinary meetings was successful. They also worked on a shared vision towards care, enhancing the connection between different professionals. They also followed training on geriatric assessments and implemented this in practice. During this year, a manager of the home care organization joined the network.
M	1 CM; 4 CN; 1 GS; 1 PP; 1 PN; 1 WF.	9 (6)	New collaboration	PN	Small	10.0; 15.5	59.2; 59.4	11; 16	Working agreements (year 1) improvement of communication (year 1) improvement of expertise (year 1)	Primary care physician was interested in the program; practice nurse took on the role as network leader. Home care, an elderly care physician and case manager responded positively to the request to join. The connection with elderly care physician and case manager needed some improvement and all professionals felt they could benefit from formulating working agreements regarding dementia care. After that, they focused on improvement of communication, of which the introduction of joint multidisciplinary care plans was one aspect. Educational sessions on problematic behavior and diagnostics were arranged. The network is stable with no changes.

Catchment area: area from which the network attracts its patient population, defined by geographical size and population distribution and density; large = more than approximately 5,000 persons.

PP = primary care physician; PN = practice nurse; CM = case manager; CN = community nurse; GS = geriatric specialist; OT = occupational therapist; PT = physiotherapist; MM = management or municipality; WF = welfare worker.

### Network maturity and quality of care

**Fig 1:**
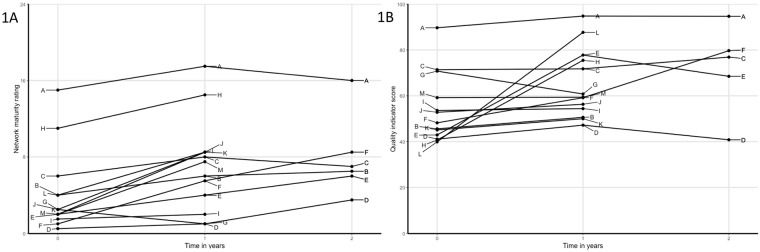
A) Trajectories of all networks over time on network maturity; B) Trajectories of all networks over time on quality of care. Networks are indicated by letters A to M and correspond with letters in Table 1.

The individual network trajectories in network maturity and QI scores over time are shown in Fig 1A and 1B. In total, 19 yearly evaluations were completed with network maturity scores, of which 16 showed improvement and 13 increased more than 2 points. The networks completed 18 evaluations with QI scores, of which 14 showed improvement (10 evaluations with an increase of over 5 points). Improvements in network maturity were accompanied by an increase in QI scores in 13 of 15 cycles.

The regression model with network maturity as dependent variable showed an estimated increase of 2.03 (95%CI 1.20–2.96) in network maturity per year in the networks. The regression model with QI scores as dependent variable showed an average increase of 8.45 (95%CI 2.80–14.69) per year in the networks. When extending the latter model by including the network maturity score as a time-varying predictor, the QI scores indicated to be positively associated with network maturity (2.11; 95%CI 0.89–3.33).

### Experiences with DementiaNet program

#### Collaboration

In general, care professionals perceived that participation resulted in shorter communication channels, higher acquaintanceship with each other’s disciplines as well as personally, increased overview of local professionals and easier access to other disciplines, such as occupational therapists and physiotherapists.

#### Care processes

Perceived impacts of DementiaNet on care were: increased and more active monitoring of individual dementia patients as well as at population level of older patients, introduction and improvement of multidisciplinary meetings, increased expertise in diagnostics subsequently resulting in more and earlier diagnoses, a shift towards diagnostics in primary care instead of referral to expert clinics, increased person-centered care regarding care needs of patients and informal caregivers, and better coordination of different care services.

#### Benefits for professionals and patients

Perceived benefits for professionals included more awareness about dementia in general and feeling more competent to care for people with dementia. Regarding the network collaboration, professionals experienced a more profound feeling of shared goals and visions, easier and more efficient collaboration among involved care professionals, and improved coordination of care. Professionals reported no disadvantages and felt that patients and their informal caregivers gained benefit from better-timed and more efficient processes regarding the diagnosis.

#### Contextual factors

Conditions that enhanced collaboration included a sufficient size of shared patient caseload, practice-based learning that transcends boundaries of individual disciplines and networks, concrete agreements about communication, and working in close proximity to other professionals, preferably in the same building. Factors to stimulate continuity of care were integration of services from different disciplines by means of multidisciplinary meetings and multidisciplinary care plans, and short communication channels between all involved care professionals (i.e. by shared infrastructure to exchange information). The presence of active and capable network leaders seemed to play a key role in achieving actual improvement goals.

### Integration

**Fig 2:**
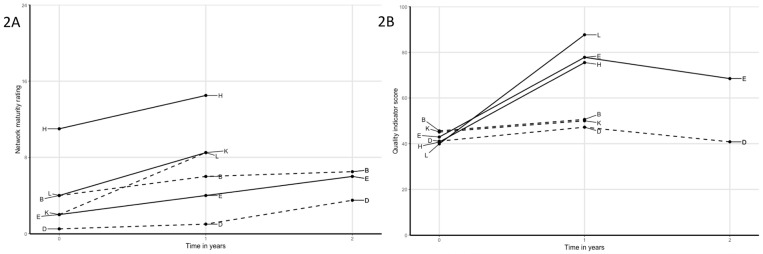
A) Network maturity trajectories of all networks; solid lines are networks with relatively low starting level of quality of care but with strong improvement; dashed lines are networks with equally low starting level of quality of care, but no susbtantial improvement. Networks with solid lines where characterized by high involvement of the primary care practice, network leaders in the primary care practice and operating in rural areas, and; B) quality of care trajectories.

**Fig 3:**
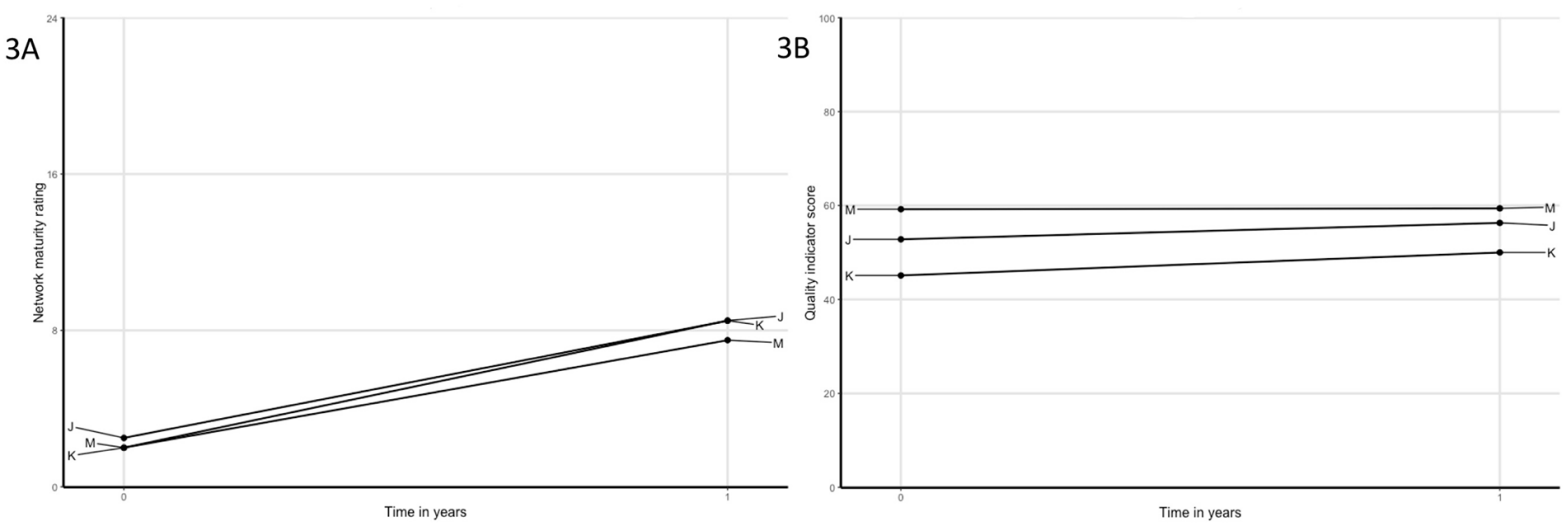
A) Network maturity trajectories of networks that have shown considerable improvement on network maturity, but no substantial improvement on quality of care, reflected in improvement goals that were focused on collaboration and network strength, and; B) quality of care trajectories.

**Fig 4:**
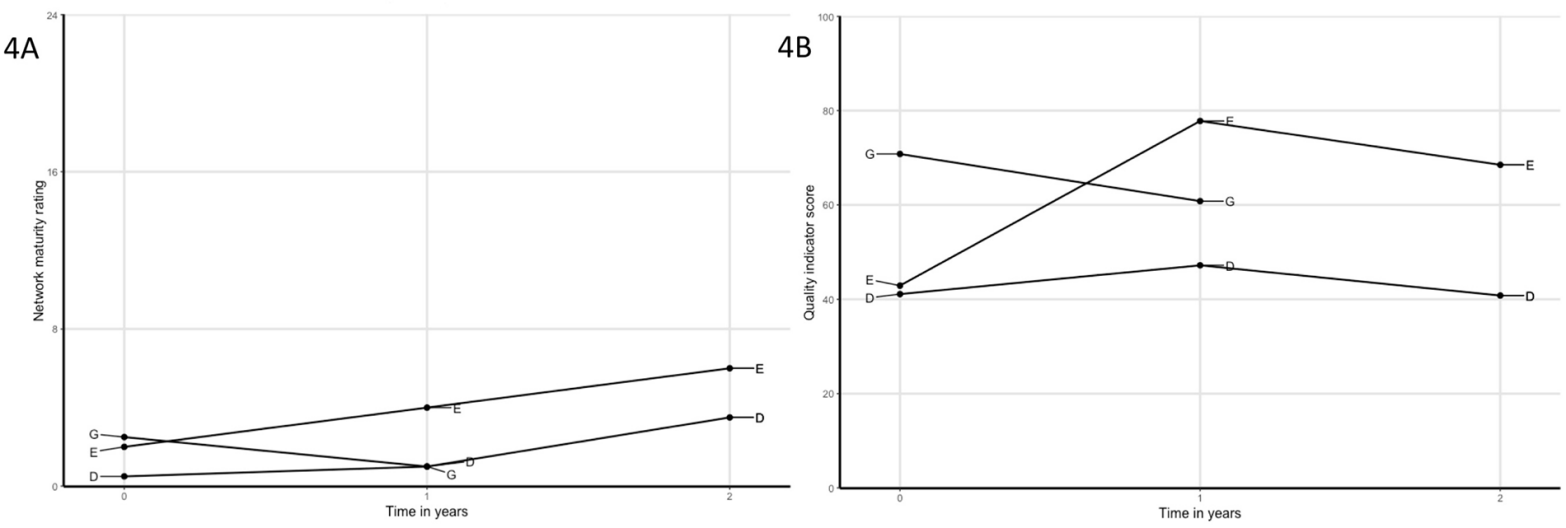
A) Network maturity trajectories of networks with decreasing quality of care scores: solid lines are networks that had various problems leading to a decrease in quality of care, and; B) quality of care trajectories.

**Fig 5:**
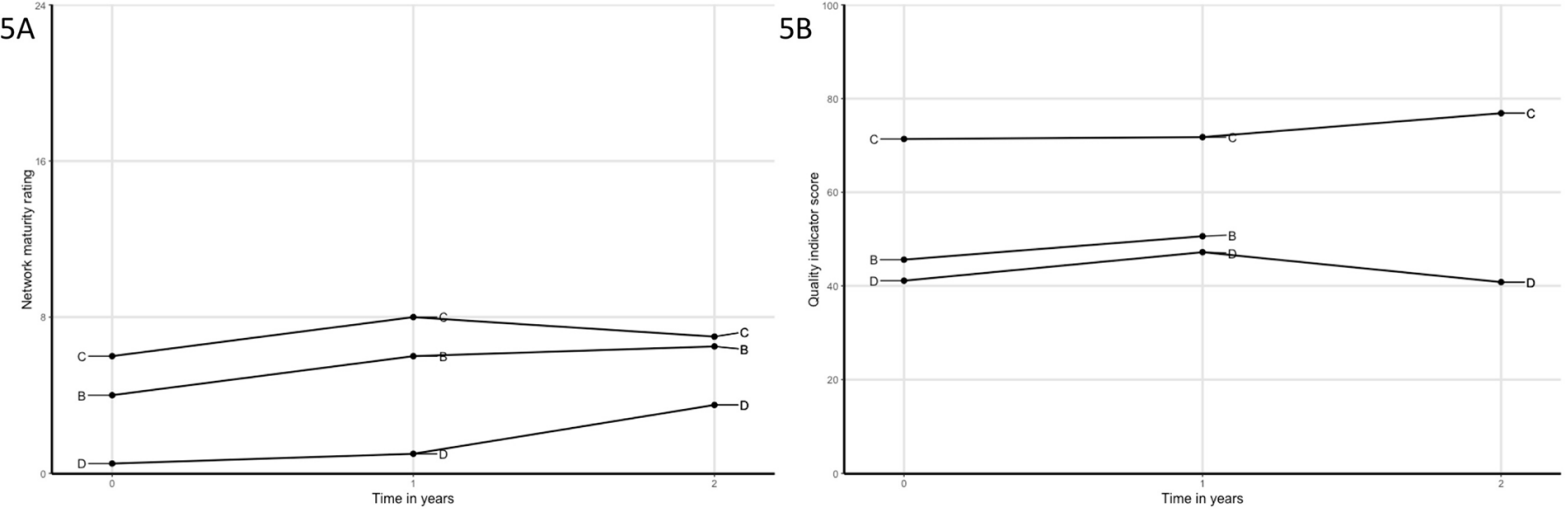
A) Network maturity trajectories of networks with suboptimal leadership and display no substantial improvement on network maturity or quality of care, and; B) quality of care trajectories.

**Fig 6:**
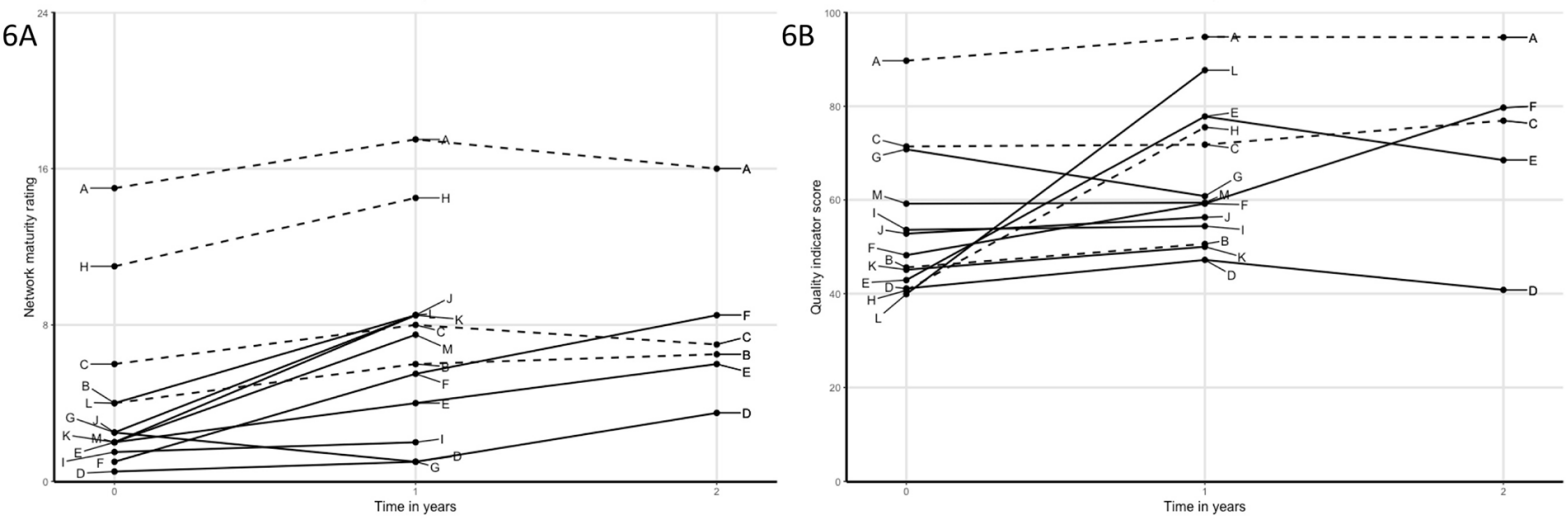
A) Network maturity trajectories of all networks; improvement goals and starting level of collaboration: dashed lines are networks with existing collaborations; solid lines are networks with new collaborations. Dashed lines indeed start at higher levels of network maturity, and; B) quality of care trajectories.

**Fig 7:**
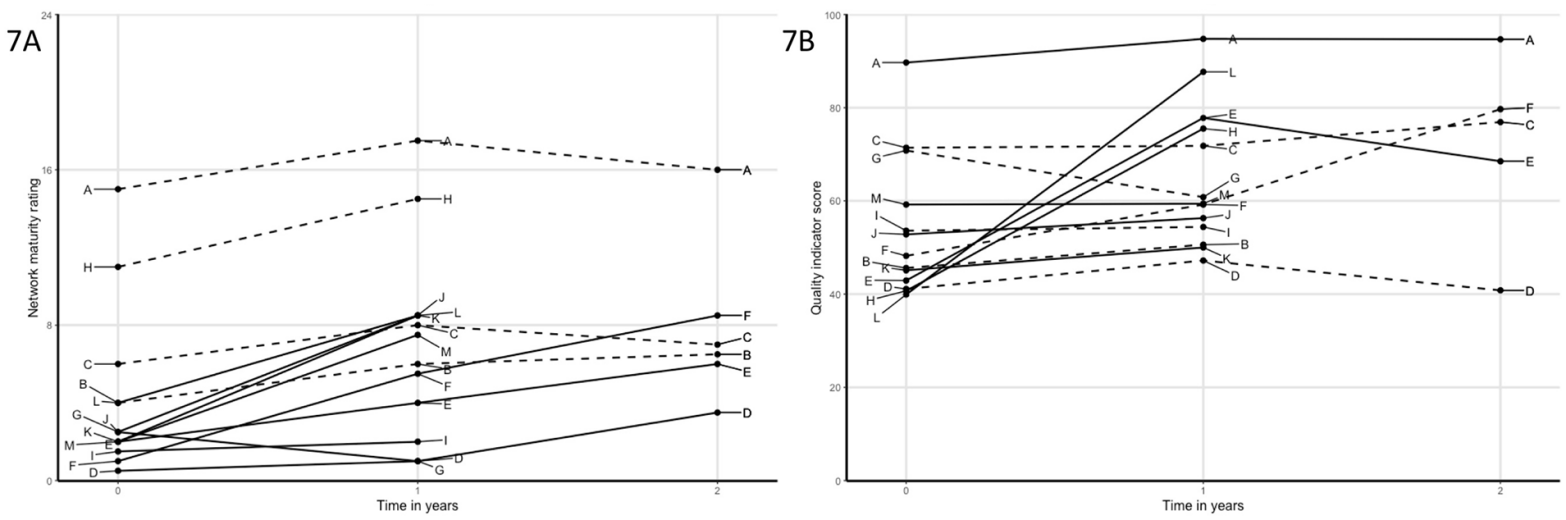
A) Network maturity trajectories of all networks; dashed lines are networks with above average catchment areas, solid lines are networks with smaller catchment areas. Solid lines show more increase than dashed lines, and; B) quality of care trajectories.

Joint interpretation of the multiple data sources led to the identification of several patterns (Table 2, Figs 2–7). Although most networks increased in network maturity, patterns showed that those networks that started with professionals who were already acquainted with each other to some extent, were more likely to increase on QI scores. Unacquainted networks were more likely to choose improvement goals focusing on initiating their network and collaboration, whereas more acquainted networks were already able to work collaboratively on actual care processes.

**Table 2:** Inferences from joint interpretation of data sources.

**Patterns prompted by quantitative findings**	
Networks starting at low quality of care	Of the six networks with the lowest QI scores at start, three showed very large increases over the first year (E, H, L) and three had only minor increases (B, D, K). Several factors may explain differences between these two groups. Most importantly, the three successful networks are characterized by active participation of primary care practice: the PPs are highly involved as team members and have an active role in the improvement plans, and network leaders all work as part of the primary care practice. Also, the successful networks are relatively small and more rural compared to the less successful networks.
Network maturity as prerequisite to increase quality of care	Networks J, K and M started as (fully) new collaboration and showed considerable increase in network maturity. Network J and K started as a fully new network. Hence, improvement actions were primarily aimed at getting to know each other in person as well as each other’s professions, tasks, competences and preferences. Network M started as a relatively new network with acquainted members. Improvement goals were aimed on process agreements and communication. In all networks, network maturity increased, yet, no considerable improvements were reached on QI scores. Hence, this indicates that a certain level of network maturity is required as prerequisite to enable networks to collaborate in improving care processes and thereby improving quality of care.
Declining quality of care	Some networks (D, E, G) showed decreased QI scores after quality improvement cycles. Network E showed a substantial increase in QI scores over the first year, but decreased over the second. In this network, divergent visions on good care caused problems in interpersonal relationships. In addition, both network leaders had been absent for part of the second year, which resulted in delays for improvement actions. Network G started without several key players, but with the intention to involve a primary care practice along the way. This was deemed unsuccessful due to several reasons: the geographical area was large including multiple different primary care practices; PPs showed no interest to collaborate or join as network members; and inability to improve care processes without involvement of relevant primary care practices. Hence, attention was largely aimed at initiating overall collaboration as a network, instead of working as a team on patient-level processes. Network D showed minor increase in the first year, but decreased to the starting level during the second year. Although advancements were accomplished during the second year, this was not reflected in the QI score. The primary improvement goal was the implementation of an online communication tool (not reflected in the QI scores). Other improvement goals received little attention and network leadership was suboptimal (one of two leaders was replaced and both felt less motivated because of little actions undertaken by other network members).
**Patterns prompted by network characteristics**	
Strength of leadership	Three networks (B, C, D) were identified based on the fact that leadership was observed to be suboptimal, with absent leaders, insufficient time investment to adequately lead improvement actions, no acceptance of the leadership role other network members, or leaders were not assertive enough for improvement plans to proceed. Indeed, networks with suboptimal leadership were not among those that displayed strong progression either on network maturity or QI scores. Networks with leaders from the primary care practice seemed to be more successful than those with other leaders.
Improvement goals and starting level of collaboration	Four networks (A, B, C, H) were characterized as existing collaborations. This corresponded with high network maturity at start. Hence, improvement cycles were not aimed at increased acquaintanceship but mainly focused at increasing dementia-specific care processes and expertise (i.e. cognitive decline, problematic behavior in patients and dementia-friendly society). Notably, improvement goals of those networks that just started collaboration and networks with lower network maturity scores at start were more often aimed at initiation or organization of collaborations, to meet each other and work together in the setting of primary dementia care.
Catchment areas	Networks were categorized as having either a small or large catchment area, depending on the size of the population (i.e. geographical area and population density) in the area they operate in. High density areas (often urban) are particularly characterized by a high variety in services available with numerous care providers (e.g. multiple home care organizations), increasing the number of professionals working in those geographical areas, decreasing the number of shared patients and an increased presence of competition, which all might complicate actual collaboration. Networks in large catchment areas (B, C, D, F, G, I) had higher average size of the networks. This also reflects more complex collaborations within the networks. With the exception of network F, the networks with the large catchment areas showed considerably less improvement, both on network maturity as on QI scores.
**Patterns prompted by success of networks**	
Best practices	Two networks can be described as “best practices”. Network A was already at an exceptionally high level, both on network maturity and QI scores. Network F was a newly started collaboration and hence started rather low, but proceeded to high scores during the course of the program. In network A, several elements have been identified making this network state of the art. First of all, they started as a tight group of professionals that have worked together for a long period. The fact that they were situated in a rather small village resulted in a limited number of professionals operating in the area, so they basically work together for all dementia patients in the area resulting in a sufficient shared caseload. Strong PP leadership and a long mutual history, ensure high levels of acquaintanceship and trust among these professionals, as well as highly structured care processes. Explicit agreements have been laid out for many processes (e.g. diagnosis and assessments). Furthermore, they have a well-structured multidisciplinary meeting to discuss each patient, which is a central aspect, with active involvement of patients and informal caregivers. The meeting results in (adjustment of) a multidisciplinary care plan, which is available to all professionals in an online infrastructure, including informal caregivers. This ensures continuity and stimulates collaboration to a great extent. Network F already had the preconditions for a mature network at enrollment, such as acquaintanceship and a history together, but had not gotten around to defining their network and the processes, partly due to lack of knowledge and leadership. Upon starting in the DementiaNet program, both needs were addressed at the very start. This network was then capable of defining a collaborative structure and simultaneously working on specific care processes, resulting in a high increase in both network maturity and quality of care scores over both years. Their major focus points were the disciplinary meeting and diagnosis. Characteristics that both networks have in common are the highly involved primary care practice, network leader(s) working in the primary care practice, and strongly basing collaboration and coordination on highly structured and frequent multidisciplinary meetings at fixed time points as the main way to communicate about individual patients.
Unsuccessful networks	The common denominator of the four networks that ceased participation within the first year is that no sufficient momentum was created to form a network. Overall, a necessary level of commitment and motivation was not reached before enrollment in the program in these networks. In one case, the network was initiated by a local government, although the participating healthcare professionals were not very motivated. In another network, there were problems with the primary care practice staff (the core of this network), and they felt like priority should be given to keeping up with regular work instead of investing in new initiatives.

The PCP was identified as an essential element of successful network-based care. Patterns showed that networks with highly involved PPs performed better than those without or with only little involvement. Especially, those networks in which leadership was assigned to staff working at the PCP (i.e. PP or practice nurse) improved. These findings were also confirmed in the two best performing practices, in which primary care practice involvement seemed to play a central role in their success.

Leadership in general was an important prerequisite for success. Networks that experienced problems with leadership and those without competent leaders showed no or only minor improvements. Furthermore, lack of accurate leadership was possibly one of the factors leading to decreases in QI scores and network maturity, along with not having all key disciplines involved in the network and interpersonal problems among network members.

The area in which networks operated seemed to influence network sizes and the magnitude of improvement: networks with larger catchment areas, on average, showed higher numbers of involved professionals, likely as a result of higher numbers of care providers operating in those catchment areas. This increases the complexity of collaboration and also decreases the shared caseload between several professionals in these networks. Networks from smaller catchment areas displayed increased progression of network maturity and QI scores.

## Discussion

Seventeen networks were successfully established of which 13 accomplished one or more active years in the DementiaNet program. Overall, this multiple case study showed an increase in both network maturity and quality of care and a positive association was indicated between these measures. Multidisciplinary collaboration, communication, and coordination of care improved according to healthcare professionals in the networks, and DementiaNet enabled them to beneficially impact care. Importantly, prerequisites for successful transition towards more mature and integrated networks were identified.

These findings indicate that most DementiaNet networks successfully transitioned towards a more mature and integrated network. The estimated overall change over time in network maturity per year in the program was approximately two points and estimated change in QI scores was approximately 8.5 points. To illustrate practical relevance, this might indicate that in a single year, a network progresses two levels on the maturity model (e.g. from ad hoc to defined professional and clinical integration). For quality of care this implicates, for example, that more patients were discussed during multidisciplinary meetings. These results show beneficial impact, even after only one active year in the program. As the yearly evaluations reflect an iterative transition process, maximal effects are not realistically expected in such a short time frame. Therefore, likely, larger effects may be expected in the long term, as major change emerges from aggregation of marginal gains.[18]

Several enabling factors for the successful transition to network-based care were identified. These factors included strong and adequate leadership (preferably with leaders from primary care practice), high involvement of motivated PPs, high acquaintanceship with other network members, and network size with a compact network that operates in a relatively small geographical area. These empirical findings corroborate with theoretical models on primary care collaboration.[17, 19]

DementiaNet was developed from a system-level perspective to fit the complexity of clinical practice. Lessons from previous successful redesign efforts have shown that it is unlikely that single stakeholders can create a highly functioning system.[18] Indeed, some previous studies have shown that programs aimed at single aspects of care (e.g. lack of expertise) have had limited to no effects on the care system.[20, 21] Following from complexity theory, this may not be surprising, as changes on multiple levels are needed to ensure change on the system as a whole.[22, 23] In line with this assumption, studies targeted at a more comprehensive level, for example case management intensity and health and social services integration, resulted in beneficial effects for dementia patients.[24] Also, other collaborative care models employing a system-wide approach such as the Aging Brain Care Medical Homes in the US and the German Delphi-MV study, have shown positive results.[2, 22, 25, 26]

The major strengths of the DementiaNet program were the simultaneous focus on various essential aspects of high-quality network-based care, the practice facilitation approach with support at local level with local leadership, and its flexibility to be modified to varying circumstances of each individual network. Also, being able to choose and set their own goals appeared to be a major advantage of the program and motivated network member to work on improvements.

Multilevel programs are needed to achieve meaningful impact on healthcare systems,[9] yet, evaluation is challenging, especially when innovations are implemented in a complex setting as daily clinical practice.[27, 28] The current evaluation study succeeded in including the flexibility and individualization of the approach, and in identifying generalizable factors between networks. Its mixed methods design allowed for consequences of complexity such as unpredictability in outcomes, by ensuring an open view.[9] Also, the interviews provided relevant insights on the process of change, and allowed to include unanticipated effects (a feature of complex systems) as well. Moreover, the multiple case study design permitted for the analysis of group effects and to simultaneously study individual networks more closely to identify mechanisms and contextual factors that stimulated or hindered change.

This study had some limitations, starting with the relatively limited follow-up. The DementiaNet approach necessitates considerable changes in behavior and practice from large numbers of actors; such adaptations require time. Integration of data revealed the pattern that relatively immature networks had to define collaborative efforts first before changes in care processes could be addressed. This underlines the importance for endured time to mature for networks, especially in those that start as fully new collaborations. Even though major changes have been observed, longer follow-up is needed to show sustained effectiveness. Another limitation is the fact that the quality indicators used in this study were newly developed. Nonetheless, the initial set of QIs was rigorously developed through multiple consensus rounds and based on existing guidelines and agreements[13], and its face validity was assessed before application in this study. Furthermore, QIs were reviewed prior to analysis for coherence, missingness and floor and ceiling effects.

These findings might well be used to inform future application of network-based approaches, like for example care for frail elderly, where similar professionals are involved. For that purpose, the achieved diversity in the networks studied (i.e. newly or existing collaboration, small and large network size and catchment areas) is a valuable property in two ways. First, these multiple different networks have ensured information based on wide diversity of healthcare professionals and local settings. Secondly, it has shown that the design of DementiaNet allows for adaptation to local complexity and individualization, which might serve as a basis for translation to other populations and various settings as well. When research findings of the current study are to be applied to other settings, the context needs to be taken into account, as this plays an important role in the success of the DementiaNet program. By providing detailed contextual information, application to other settings is facilitated.

To conclude, the DementiaNet program resulted in a successful transition towards more integration in primary care networks, which was accompanied by an overall increase in quality of dementia care. Collaboration between network members from different disciplines and coordination of care improved. Explanatory mechanisms were involvement of the primary care practice, strong leadership and network catchment area and size. These transitions appear to benefit patients and informal caregivers, as well as primary care professionals. The use of a longitudinal mixed methods multiple case study revealed a complete and integrated picture of effectiveness, and can therefore contribute to the increased use of innovative research designs for the future evaluation of complex interventions.
